# *BdorOR88a* Modulates the Responsiveness to Methyl Eugenol in Mature Males of *Bactrocera dorsalis* (Hendel)

**DOI:** 10.3389/fphys.2018.00987

**Published:** 2018-07-26

**Authors:** Huan Liu, Zheng-Shi Chen, Dong-Ju Zhang, Yong-Yue Lu

**Affiliations:** Department of Entomology, South China Agricultural University, Guangzhou, China

**Keywords:** *Bactrocera dorsalis*, methyl eugenol, transcriptomic analysis, olfactory, odorant receptor, *Xenopus* oocytes

## Abstract

Insect attractants are important prevention tools for managing populations of the Oriental fruit fly, *Bactrocera dorsalis* (Hendel), which is a highly destructive agricultural pest with health implications in tropical and subtropical countries. Methyl eugenol (ME) is still considered the gold standard of *B. dorsalis* attractants. Mature male flies use their olfactory system to detect ME, but the molecular mechanism underlying their olfactory detection of ME largely remains unclear. Here, we showed that ME activates the odorant receptors *OR63a-1* and *OR88a* in mature *B. dorsalis* males antennae by RNA-Seq and qRT-PCR analysis. Interestingly, ME only elicited robust responses in the *BdorOR88a*/*BdorOrco*-expressing *Xenopus* oocytes, thus suggesting that *BdorOR88a* is necessary for ME reception and tropism in *B. dorsalis*. Next, our indoor behavioral assays demonstrated that *BdorOR63a-1* knockdown had no significant effects on ME detection and tropism. By contrast, reducing the *BdorOR88a* transcript levels led to a significant decrease in the males’ responsiveness to ME. Taken together, our results gave novel insight in the understanding of the olfactory background to the Oriental fruit fly’s attraction toward ME.

## Introduction

Insects rely primarily on sophisticated olfactory reception systems to detect and discriminate many exogenous chemical signals, and odorant receptors (ORs) are at the core of odorant detection (Hallem and Carlson, 2006; Missbach et al., 2014; Zhang et al., 2016; Fleischer et al., 2017; Wang et al., 2017). Apparently, ORs show a high degree of sensitive and play critical roles in detecting long-range volatile odorants and triggering the transduction of chemical signals into electric signals (Mitsuno et al., 2008; Leal, 2013). For example, an odorant receptor isolated from *Plutella xylostella*, *PxylOR1*, was narrowly tuned to the main component of the sex pheromone, (11*Z*)-hexadecenal (Z11-16Ald) (Sakurai et al., 2011). Interestingly, the direct activation of *CquiOR136*, is necessary for DEET (*N*,*N*-diethyl-3-methylbenzamide) reception and repellency effects in *Culex quinquefasciatus* (Xu et al., 2014). Additionally, knockdown of *AjapOR35* in *Anastatus japonicus* reduced its antennal response to two oviposition attractants, β-caryophyllene and (E)-α-farnesene (Wang et al., 2017). In this respect, using a “computational reverse chemical ecology" strategy will likely generate new insights for the rapid screening of potentially effective semiochemicals that modify the behavioral patterns of insects (Siderhurst and Jang, 2006; Wu et al., 2015).

The oriental fruit fly, *Bactrocera dorsalis* (Hendel), is among the most destructive fruit/vegetable-eating agricultural pests in the world (Zheng et al., 2013; Liu et al., 2016). Due to polyphagia in the larval stages and high fecundity of the adults, *B. dorsalis* can cause serious damage to more than 250 species of commercially grown vegetables and fruits (Clarke et al., 2005; Stephens et al., 2007; Shen et al., 2012). Plant damage caused by *B. dorsalis* consists of oviposition stings to host fruit tissues by adult females and the subsequent larval feeding and decaying in the fruit pulp. Controlling the male adult fly’s behavior is the main method to reduce the damage caused by this pest (Clarke et al., 2005). Methyl eugenol (ME), a highly potent phytochemical lure, has been exploited in the male annihilation technique (MAT) systems for detecting, monitoring, and luring *B. dorsalis* male individuals (Vargas and Prokopy, 2006; Pagadala et al., 2012; Shelly, 2017). Particularly noteworthy is the fact that ME has its risks, however: it is carcinogenic to humans and its attractiveness is limited to mature males (Smith et al., 2002; Khrimian et al., 2009; Zheng et al., 2012). Although novel attractants of *B. dorsalis* are diligently being developed, their progress toward better and more affordable attractants has nonetheless been slow (Khrimian et al., 1994, 2006, 2009; Jang et al., 2011). Design of attractants to target specific ORs may promote the development of new baits for pest management (Di et al., 2017; Mitchell et al., 2017). However, ME receptors ORs in *B. dorsalis* are hitherto unknown. Therefore, exploring the molecular mechanism underlying olfactory detection of ME in *B. dorsalis* is of great importance for developing sustainable pest control strategies based on manipulating insect chemosensory communication.

In trying to identify the chemosensory genes responsible for detecting ME, recent studies have identified and confirmed that *BdorOrco*, *BdorOBP83a-2*, and *BdorOBP2* actively participate in the process of ME detection by *B. dorsalis* male adults (Zheng et al., 2012; Wu et al., 2016; Liu H. et al., 2017). Nevertheless, the specific ORs and their molecular functional involvement in the mature male fly’s reception of ME remain a mystery. Here, we used an RNA-Seq approach coupled to RNA interference silencing, supplemented by *in vivo* expression in *Xenopus laevis* oocytes, to investigate the functional roles of *BdorOR63a-1* and *BdorOR88a*; both genes are abundantly expressed in ME-treated male antennae and were hypothesized to encode possible receptors for ME. Based on the results presented here, we infer that *Bdor*OR88a likely plays an essential role in the molecular mechanism underlying *B. dorsalis* olfactory detection of ME.

## Materials and Methods

### Ethics Statement

No specific permits were required in our studies of this widespread agriculture pest. We confirm that the study locations were not privately owned or protected. This work did not involve endangered or protected species. To avoid chemical hazards, always observe safety laboratory practice when operating the ME.

### Insect Rearing

The *B. dorsalis* genetic sexing strain (GSS) used in this study was reared in a laboratory for more than 30 generations at the South China Agricultural University, Guangzhou, China. The male pupae are brown and the female pupae are white. Insects were reared under a photoperiod cycle of 14 h light/10 h dark at 27 ± 1°C, 75 ± 1% relative humidity (RH). Larvae were reared on an artificial diet that included yeast (15.06%), sugar (8.99%), nipagen (0.15%), sodium benzoate (0.15%), citric acid (1.70%), wheat germ oil (0.15%), and water (73.81%) (Chang et al., 2006; Liu H. et al., 2017). Adult flies were maintained in 35 cm × 35 cm × 35 cm wooden cages and fed a diet consisting of sugar: yeast extract at 1:1 (w/w) (Liu H. et al., 2017).

### Treatments and Samplings

The ME solution used consisted of 1:1 dilution with mineral oil (MO) (Energy Chemical Company, Shanghai, China), of which 0.5-ml amounts were used to coat the inner wall of 500-ml conical flasks. Next, sample of 200 mature males (15-day-old) were randomly selected and placed in each flask. Flies in the control group were likewise handled but put into flasks containing an equal volume of MO only. After being treated for 1 h, all the antennae were dissected and flash frozen in liquid nitrogen, and were then immediately transferred to a -80°C freezer pending RNA extraction. Three independent biological replicates were performed for use in the transcriptome analyses. Experiments were conducted between 9 and 11 a.m. During these experiments, RH and temperature in the laboratory were maintained at 75 ± 1% RH and 27 ± 1°C, respectively.

### RNA Preparation, Library Construction, and Transcriptome Sequencing

Under an RNA-free environment, antennal total RNAs were extracted by using a RNA extraction kit (Takara Biotechnology Co., Ltd., Japan), following the manufacturers protocol. Each sample consisted of 200 males’ antennae. The purity of all RNA samples was assessed at absorbance ratios of OD_260/230_ and OD_260/280_, while the integrity of RNA was verified through 1%-RNase-free agarose gel electrophoresis. The concentration of RNA was quantified by measuring their absorbance at 260 nm in a spectrophotometer (Thermo Nano Drop^TM^ 2000c; Santa Clara, CA, United States) and qualified using an Agilent 2,100 Bioanalyzer (Agilent Technologies, Inc., Santa Clara, CA, United States). High-quality RNA from each replication of the ME treatment and control groups was used for the next suite of steps: mRNA was first isolated from total RNA using magnetic beads with oligo (dT) and sheared into short fragments in a fragmentation buffer. Then complementary DNA (cDNA) was synthesized from the mRNA fragments, by using SuperScript III Reverse Transcriptase (Invitrogen). A cDNA library was constructed for each sample. The library preparations were sequenced on an Illumina HiSeq 4000TM platform (Illumina Inc., San Diego, CA, United States) with 125-nucleotide (nt) paired-end reads; this final step carried out by the Gene Denovo Technology Company (in Guangzhou, China).

### Sequence *de Novo* Assembly and Functional Annotation of Unigenes

For quality control, the raw sequencing data in the FASTQ format were processed by in-house Perl scripts to obtain clean data reads. Before assembly, any adapter sequences were removed from the raw reads. Short or low-quality reads – those reads containing an adaptor, reads containing >5% unknown nt “N,” and reads with >20% quality value ≤10 – were removed from raw data to obtain more reliable results. The Q20, Q30, and GC contents of the cleaned data were calculated. Next, the clean reads were *de novo*-assembled by using Trinity software (version v2013-02-25) (Trinity Software, Inc., Plymouth, MA, United States) and clustered with TGICL Clustering tools (Version 2.1) (The Institute for Genomic Research, Rockville, MD, United States) (Pertea et al., 2003; Grabherr et al., 2011). Functional annotation of these assembled unigenes was performed with BLASTx^1^, an integrated Gene Ontology (GO) annotation and data mining tool that assigns GO based on four publically available databases: the National Center for Biotechnology Information (NCBI) non-redundant protein database (Nr), the Kyoto Encyclopedia of Genes and Genomes (KEGG^2^), the Eukaryotic Ortholog Groups (KOG^3^), and the Swiss-Prot protein database^4^. All searches were performed with an *E*-value < 10^-5^. We used the Blast2GO^5^ program to do the GO functional classification for all unigenes, according to their molecular function, biological process, and cellular component (Ashburner et al., 2000; Conesa et al., 2005).

### Differential Expression Analysis of Genes

The sequenced reads for each sample were remapped onto the reference sequences with RSEM software v1.2.12 (Li et al., 2011; Zhang et al., 2016). Gene expression levels were estimated using the Fragments Per Kilobase of transcript per Million fragments (FPKM) method that is based on the number of uniquely mapped reads (Trapnell et al., 2010). Genes differentially expressed between the male antennae from ME treatment and MO groups were identified based on their FPKM values (Mortazavi et al., 2008; Anders and Huber, 2010). The false discovery rate (FDR) adjustment was made to correct the *P*-value threshold in these multiple tests and analyses (Benjamini and Hochberg, 1995). An FDR-adjusted *P*-value < 0.05 and an absolute value of the log_2_ ratio > 1 were set a priori as the significance threshold for gene differential expression in this study. For convenience, the differential expression genes showing higher expression levels in the ME than in the MO group were designated as “up-regulated,” whereas those displaying lower expression levels were designated as “down-regulated.”

### Functional Analysis of Differentially Expressed Genes (DEGs)

Differentially expressed genes were also annotated using the GO database, and the numbers of DEGs in each GO term were calculated. To determine, which GO terms were significantly enriched in the DEGs, we performed a GO enrichment analysis that used a hypergeometric test to map all differentially expressed proteins to the GO terms in the database. This test used the following equation (Blüthgen et al., 2005; Tu et al., 2015):

$$P=1-\sum_{i=1}^{m-1}\frac{\left({}_{i}^{\begin{array}{l} M \end{array}}\right)\left({}_{n-i}^{N-M}\right)}{\left({}_{n}^{N}\right)}$$

where, *N* is the number of all genes with a GO annotation; *n* is the number of DEGs in *N*; *M* is the number of all genes annotated to specific GO terms; and *m* is the number of DEGs in *M* (M–m ≥ 0). The calculated *P*-value was first subjected to a Bonferroni correction, taking a corrected *P*-value of 0.05 as a threshold for statistical significance. GO terms fulfilling this condition were defined as significantly enriched GO terms in the DEGs.

All identified genes were mapped to pathways in the KEGG database by using the KEGG Automatic Annotation Server software. To identify significantly enriched metabolic pathways or signal transduction pathways in DEGs, we used the same formula calculation as in the GO analysis. Here, *N* represented the number of all genes with a KEGG annotation, *n* is the number of DEGs in *N*, *M* is the number of all genes annotated to specific pathways, and *m* is the number of DEGs in *M*.

### Gene Expression Validation by Quantitative Real-Time PCR (qRT-PCR)

To verify our RNA-Seq results, 16 genes related to insect olfactory transport process that showed different expression levels, as revealed via RNA sequencing, were randomly selected for validation in a qRT-PCR analysis. In addition, other independent sampling experiments were conducted in order to obtain new biological replicates by ME and MO treatment. Those experiments were performed as described above. A RNA extraction kit (Takara Biotechnology Co., Ltd., Japan) was used to extract antennal total RNA from the ME treatment and control groups of male flies according to the manufacturer’s protocol, and a gDNA eliminator spin column removed genomic DNA. Approximately 1 μg of total RNA from each sample was used to synthesize cDNA, by using a PrimeScript^TM^ RT Reagent Kit (Takara Biotechnology Co., Ltd., Japan), which then served as a template for qRT-PCR. The gene-specific primers were designed according to the gene sequences in Primer v5.0 software (Premier, Canada) were listed in Supplementary Table S1. RT-PCR was performed to test whether all primers could amplify the correct products. Amplification efficiencies of all primers were validated before the gene expression analysis.

To perform the qRT-PCR reactions, a SYBR Premix ExTaq Kit (Tiangen, Guangzhou, China) was used following the manufacturer’s instructions, and run on a Stratagene Mx3000P thermal cycler (Agilent Technologies, Wilmington, Germany). qRT-PCR was carried out according to the protocol reported in our previous study (Liu H. et al., 2017). The α*-tubulin* gene of *B. dorsalis* was amplified to serve as the internal control (GenBank accession number: XM_011212814) (Shen et al., 2010; Yang et al., 2013; Liu et al., 2015; Gui et al., 2016). Dissociation curve analyses were performed to ensure amplification specificity. Three independent biological and three technical replicates were used and performed for each gene, respectively. The relative gene expression levels were calculated by using the 2^-ΔΔC_T_^ method as described previously (refer to Livak and Schmittgen, 2001).

### Sequence Analysis of *BdorOR63a-1* and *BdorOR88a*

To identify the characteristics of *B. dorsalis* OR genes, particularly the significant differently expression ORs (*BdorOR63a-1*, GenBank accession number: KP743726 and *BdorOR88a*, GenBank accession number: KP743732), and their relationship to other Dipteran insects. To do this, a maximum likelihood tree for the ORs was constructed using the amino acid sequences derived from *B. dorsalis* and the published sequences of two Dipteran species: *Ceratitis capitata* (Wiedemann) and *Drosophila melanogaster* (Meigen). All the information on the amino acid sequences of ORs was obtained from the NCBI database. Alignments of OR amino acid sequences were performed using the program ClustalW2. The maximum likelihood tree was constructed in MEGA v7.0 software (Molecular Evolutionary Genetics Analysis, v4.0, Sudhir Kumar, United States) and with the Interactive Tree Of Life (iTOL) web tool^6^.

### Effects of Age and Daily-Rhythm on the Male Fly Responsiveness to ME and the *BdorOR63a-1*, *BdorOR88a* Expression Levels

Bioassays were performed in the laboratory following a method similar to that of Karunaratne and Karunaratne (2012) and Liu H. et al. (2017). For the assessment of effects of age on the male responses to ME, each sample of 2-day- and 10-day-old males (200 of each) were randomly selected as the test subjects and released into a screened cage (1.0 m × 1.0 m × 1.0 m) without a trap. Approximately 30 min later, a fly trap containing 1.0 mL of pure ME was placed inside the screened cage. For the control, a trap was likewise placed inside the cage but without any ME. After trapping for 2 h, we removed the traps and counted the number of attracted flies. This bioassay was conducted between 9 and 12 a.m. under daylight conditions. Three independent biological replicates were performed.

To determine whether the mature male response to ME varied throughout the day, the responses of 10-day-old male *B. dorsalis* were assayed at 9 a.m., 1 p.m., and 5 p.m., as described above. For this, three biological replicates of flies were used per ME treatment and per control group for each time point. Additionally, from the untested individuals, the antennae from the 2-day- and 10-day-old males, and the mature males at 9 a.m. (morning), 1 p.m. (early afternoon), and 5 p.m. (near dusk), were dissected and immediately frozen in liquid nitrogen. Then, their total RNAs were extracted and reverse transcribed into single-chain cDNAs. Next, the expression of *BdorOR63a-1* and *BdorOR88a* was evaluated by qRT-PCR quantitative technique. Each treatment was replicated three times.

### Expression of *BdorOR88a* and *BdorOR63a-1* in *Xenopus laevis* Oocytes and Two-Electrode Voltage-Clamp Electrophysiological Recordings

*BdorOR88a*, *BdorOR63a-1*, and *BdorOrco* (GenBank accession number: EU621792) were amplified using specific primers (Supplementary Table S2). The purified PCR products were ligated into the pCS2^+^ vector; then, linearized modified pCS2^+^ vectors were used to synthesize cRNAs by using the mMESSAGE mMACHINE SP6 Kit (Ambion, Austin, TX, United States) following the manufacturer’s instructions. The purified cRNAs were re-suspended in nuclease-free water at 2000 ng/μL.

The oocyte microinjection and two-electrode voltage clamp recording were performed following published protocols (see Xu et al., 2014; Li et al., 2017; Liu F. et al., 2017). Briefly, mature healthy *X. laevis* oocytes (stage V–VII) were treated with 2 mg/ml of collagenase I (GIBCO, Carlsbad, CA, United States) in a washing buffer (96 mM NaCl, 5 mM MgCl2, 2 mM KCl, and 5 mM HEPES [pH = 7.6]) for ca. 1 h at room temperature. Next, the oocytes were microinjected with 27.6 ng of *BdorOR* cRNAs and 27.6 ng of *BdorOrco* cRNA, and then incubated at 18°C for 3–8 days in 1 × Ringer’s solution (96 mM NaCl, 5 mM MgCl2, 5 mM HEPES, 2 mM KCl, and 0.8 mM CaCl2 [pH = 7.6]). Stock solutions (1 M) of ME and MO were prepared in DMSO and they were subsequently diluted to the indicated concentrations with 1 × Ringer’s buffer. The two-electrode voltage-clamp (TEVC) technique measured the odorant-induced currents in *Xenopus oocytes.* Whole-cell currents were recorded and amplified by an OC-725C amplifier (Warner Instruments, Hamden, CT, United States) at a holding potential of -80 mV, low-pass-filtered at 50 Hz, and digitized at 1 kHz. Oocytes with nuclease-free water injection served as the negative control. Data acquisition and analysis were conducted with Digidata 1440A and pCLAMP10 software (Axon Instruments Inc., Union City, CA, United States).

### RNA Interference Bioassays

An RNA interference experiment was performed to demonstrate the roles of *BdorOR88a* and *BdorOR63a-1* in ME detection by male flies. To prepare the double-stranded RNA (dsRNA), we used a template cDNA generated by PCR-targeting fragments. Those primers with T7 promoter sequences used to synthesize dsRNA are listed in Supplementary Table S3. The GFP gene served as the control dsRNA (ds*GFP*) (GenBank accession number: AHE38523). Based on the manufacturer’s protocol, the dsRNAs were synthesized and purified by the MEGAscript^®^ RNAi Kit (Thermo Fisher Scientific, United States); then, their concentrations were quantified on a Nanodrop 1,000 (Thermo Fisher Scientific, United States) and their integrity was determined by 2%-agarose gel electrophoresis.

Subsequently, expression of the dsRNAs and the injection procedure for the male flies were carried out following established techniques (Liu et al., 2015; Dong et al., 2016; Hou et al., 2017; Liu H. et al., 2017). Each sample of 50 mature males (15-day-old) was randomly selected and placed into a 35 cm × 35 cm × 35 cm cage. Needles were prepared with a puller at 60°C (PC-10, Narishige, Tokyo Japan). Microinjection was performed using an Eppendorf Microinjector (Eppendorf Ltd., Germany). The injection condition was set to an injection pressure (Pi) of 600 hPa and a timing setting (Ti) of 0.6 s. For each treated male fly, 400 nL of ds*BdorOR88a* or ds*BdorOR63a-1* (2,000 ng/μL) was injected into its body cavity between the second and third abdominal segments. Males were injected with an equal volume of ds*GFP* served as negative control groups. The blank control group consisted of male flies that were fed normally. Injected and non-injected flies were reared on the artificial diet in the cages as described above. The respective numbers of dead files were counted after treatment for 24 h and 48 h. For the bioassay, males from the ds*BdorOR88a* or ds*BdorOR63a-1* treatment groups, the ds*GFP* treatment group, and the blank control group, were placed separately inside a 1.0 m × 1.0 m × 1.0 m screen cage equipped with ME as a trap. The lured males were counted after 2 h, and the silencing efficiency of *BdorOR88a* or *BdorOR63a-1* was detected by qRT-PCR. Each bioassay experiment was replicated five times.

### Statistical Analyses

All analyses were carried out by SAS v9.20 software (SAS Institute Inc. Cary, NC, United States). Results from the experimental replicates were expressed as the mean ± SE. The responses of immature and mature male flies to ME, diurnal pattern of mature male responsiveness to ME, and the expression pattern of ORs were analyzed by independent Student’s *t-*test (*P* = 0.05). Cases of *P*-values < 0.05 were considered to be statistically significant. Datasets of the attractiveness of ME to mature male, adult mortality, and gene silencing efficiencies were checked for normality of distribution and homogeneity of variances with Shapiro-Wilk’s and Levene’s tests, respectively. If data were normally distributed and had similar variances, then the means were compared by one-way analysis of variance (ANOVA). Following a significant ANOVA result, multiple comparisons among five groups were assessed by Duncan’s multiple range test (DMRT, *P* = 0.05). Non-normally distributed data were analyzed with the non-parametric Kruskal–Wallis test to compare medians (*P* = 0.05), followed by a Mann–Whitney test for follow-up pairwise comparisons. All results were plotted with Origin v9.0 software.

## Results

### Transcriptome Sequencing and Analysis

The RNA taken from the ME-treated and control (MO) males was used for RNA-Seq – with three experimental replicates per treatment – generated 23,552,399,343 raw reads. In general, all the libraries were of good quality, with average Q20 and Q30 percentages of over 95.60 and 89.41%, respectively. After removing the low-quality reads and trimming the adapter sequences, 157,514,454 clean reads were obtained for sequencing from the six samples (**Tables 1**, **2**). These clean reads were ultimately assembled into 36,215 unigenes that had a mean length of 1,147 bp, an N50 of 2,362 bp, and a GC content of 41.61% (**Table 2**). The transcriptome data were deposited into the NCBI Short Reads Archive (SRA) database under the accession number SRP124917.

**Table 1:** Statistical raw sequencing data of the RNA-Seq reads for the examined samples.

Group name	Mineral oil (control)			Methyl eugenol treatment		
	CK1	CK2	CK3	T1	T2	T3
No. of raw reads	28,225,798	26,037,232	24,184,396	28,199,572	28,483,296	28,629,132
No. of clean reads	27,061,444	25,131,852	23,175,938	27,289,334	27,449,152	27,406,734
Adapter (%)	0.05	0.05	0.06	0.08	0.07	0.06
GC content (%)	42.81	41.90	41.50	41.28	41.36	40.81
Q20 (%)	95.68	95.89	95.67	96.02	95.81	95.60
Q30 (%)	89.53	89.92	89.52	90.22	89.77	89.41

*CK1, CK2, and CK3 are repeats of the mineral oil (control) group; T1, T2, and T3 are repeats of the methyl eugenol treatment. Q20: percentage of bases for which the Phred value is >20; Q30: percentage of bases for which the Phred value is >30.*

**Table 2:** Statistics for the resulting assembled sequences.

Group name	Number
Total nucleotides (nt)	23,552,399,343
Total clean reads	157,514,454
Total assembled bases	41,553,801
Total No. of unigenes	36,215
GC percentage (%)	41.61
Unigene N50 (bp)	2,362
Maximum unigene length (bp)	27,401
Minimum unigene length (bp)	201
Average unigene length (bp)	1,147

*Unigene N50 is the length at which the accumulated length value is greater than 50% of the total lengths after ranking the unigenes from shortest to longest.*

### Identification of Differentially Expressed Antennal Genes Between the ME and MO Treatment Male Flies

**FIGURE 1:**
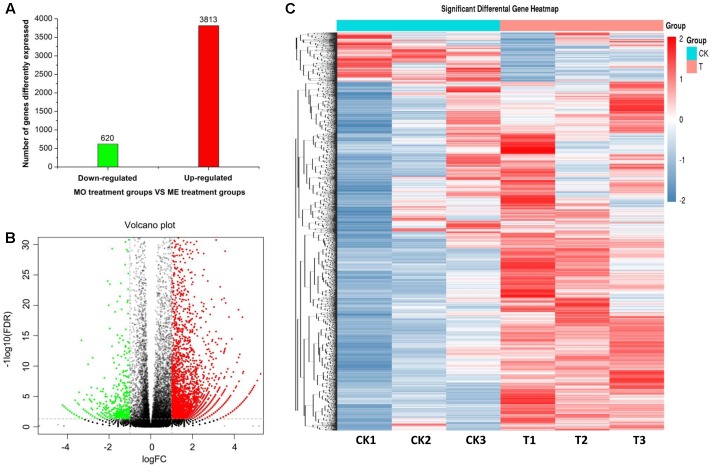
Statistical analysis of the differential expression of genes in male antennae of *Bactrocera dorsalis* flies from the MO (mineral oil) and ME (methyl eugenol) treatment groups. **(A)** Classification for the differential abundance of genes. **(B)** Volcano plots of differentially expressed genes (DEGs) between the ME and MO treatments. Y axis represents -log10 significance. X axis represents logFC (fold change). Red points indicate up-regulated expression of DEGs; green points indicate down-regulated expression of DEGs; and black points show non-differentially expressed genes. Genes with a *P*-value < 0.05 and fold change ≥1.0 were considered as DEGs in this study. **(C)** Hierarchical clustering graph of differential gene expression profiles in the ME and MO treatments. Clustering was done using RNA-Seq data derived from the six samples based on log_10_FPKM values. The column and row indicate the sample and gene, respectively. CK and T represent MO- and ME-treatment, respectively. Red and blue bands indicate, respectively, those genes that were significantly up-regulated and down-regulated in the ME-treated males.

To determine the effects of ME exposure on antennal gene expression in male *B. dorsalis* adults, their DEGs were identified using the FPKM method. A total of 4,433 DEGs were detected from the ME treatment and MO (control) groups (|log_2_FC| > 1, *P*-value < 0.05; FDR < 0.05). Of these, 3,813 (86.01%) DEGs were up-regulated and 620 (13.99%) were down-regulated in the ME-treated male antennae (**Figure 1A**). The global expression changes of all genes with RNA-Seq ratios are shown in **Figure 1B**; the green and red circles are genes having a differential expression pattern in ME-treated males compared with the control (MO group). **Figure 1C** shows the hierarchical clustering analysis of their 4,433 DEGs.

### GO and KEGG Pathway Enrichment Analysis of DEGs

With the GO annotation results in hand, the DEGs were classified and categorized into 83 functional groups (Supplementary Figure S1). Specifically, the GO enrichment analysis (*P*-value < 0.05) revealed that the most enriched biological process terms were cellular, followed by metabolic, single-organism, biological regulation, and response to stimulus; while binding and catalytic activity were the most enriched terms for the molecular function category; finally, under the cellular component category, cell and cell part were the most enriched terms (Supplementary Figure S2A). To investigate the biological pathways actively involved in ME detection by male flies, the DEGs were assigned to reference canonical pathways in KEGG: this revealed 20 significantly enriched pathways (Supplementary Figure S2B). The annotations presented here provide a valuable information for investigating the specific processes, pathways, and functions involved in the ME detection process in *B. dorsalis*, and perhaps other Diptera, too.

### Identification of Key DEGs Potentially Involved in Olfactory Function

**FIGURE 2:**
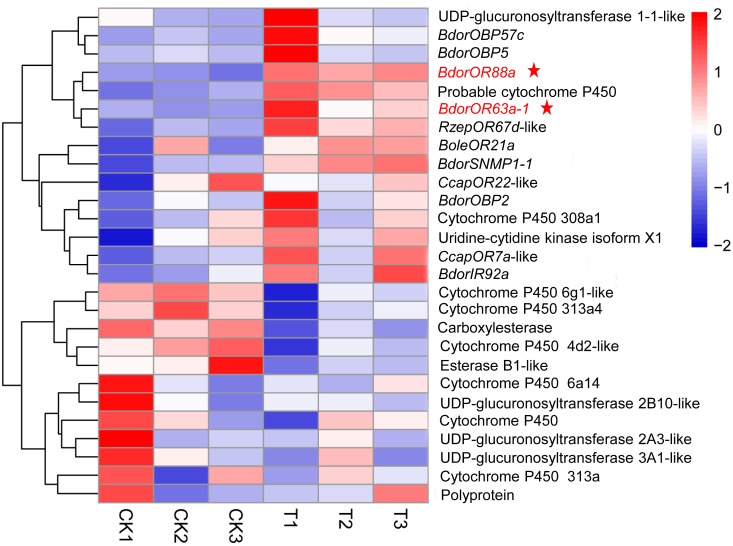
Heatmap representing the expression patterns of DEGs related to olfactory transport in male antennae from the ME (methyl eugenol) and MO (mineral oil) treatment groups. Clustering was done using RNA-Seq data derived from the six samples based on log_10_FPKM values. The column and row indicate the sample and gene, respectively. CK and T represent MO- and ME-treatment, respectively. Blue, white, and red indicate low, medium, and high levels of gene expression, respectively. Three biological replicates were conducted for each treatment.

Many DEGs associated with response to stimulus, catalytic activity, binding, biological adhesion, molecular transducer activity, and transporter activity may contribute to ME detection in adult *B. dorsalis* males. Based on the literature and our GO/KEGG enrichment analyses, we identified putative DEGs encoding proteins involved in insect olfactory transport, viz: three DEGs for odorant binding proteins (*BdorOBP57c*, *BdorOBP5*, and *BdorOBP2*), two for ORs (*BdorOR88a* and *BdorOR63a-1*), one encoding an ionotropic receptor (*BdorIR92a*), and one encoding a sensory neuron membrane protein (*BdorSNMP1-1*) (**Figure 2**). Notably, relative to the control (MO) group, both *BdorOR88a* and *BdorOR63a-1* were up-regulated by 6.33- and 2.06-fold, respectively, in the antennae of males exposed to ME. Additionally, the expression levels of *carboxylesterase*, *esterase B1*, *cytochrome P450-6a14,-313a*, and *UDP-glucuronosyltransferase 2A3, 3A1* – genes that encode candidate odorant-degrading enzymes (ODEs) – were significantly down-regulated at the transcriptional level.

### Validation of DEGs by qRT-PCR

**FIGURE 3:**
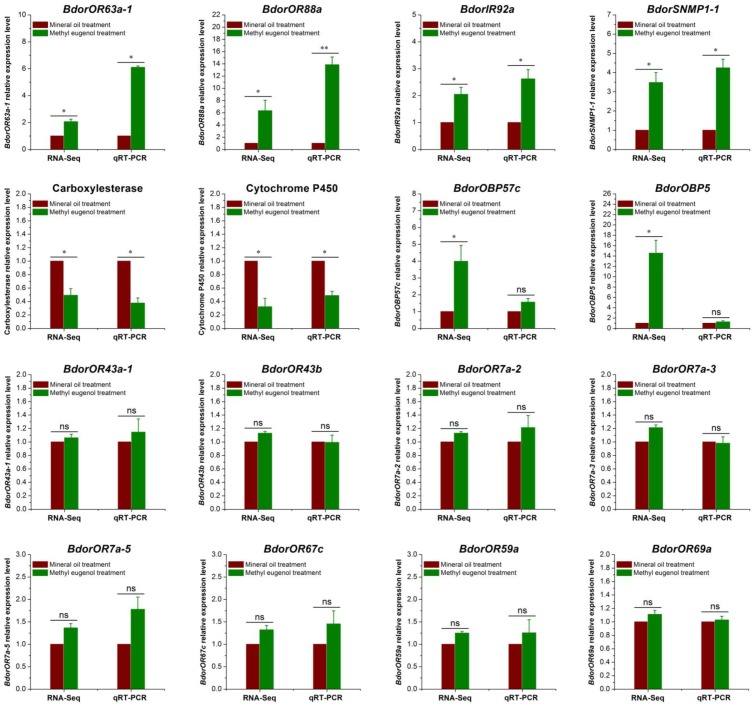
Validation of antennal DEGs (differentially expressed genes) in *Bactrocera dorsalis* male flies using quantitative real-time PCR (qRT-PCR). The α*-tubulin* gene was used as an internal control and three biological replicates were performed. The results were evaluated using a 2^-ΔΔC_T_^ method, and the 2^-ΔΔC_T_^ value of calibrant equals to 1.0. Bars represent mean ± SE values. ns, not significant, ^∗^*P* < 0.05, ^∗∗^*P <* 0.01 (*t*-tests).

We used qRT-PCR analysis to validate the results of differential gene expression obtained from the RNA-sequencing data (**Figure 3**). Two genes, *BdorOBP57c* and *BdorOBP5* expressed no significant difference in qRT-PCR analysis, which were inconsistent with RNA-Seq results. However, of the 16 selected genes, 14 agreed with our RNA-Seq results. For example, *BdorOR88a*, *BdorOR63a-1*, *BdorIR92a*, and *BdorSNMP1-1* were all significantly up-regulated in the ME-treated male antennae, as found in the RNA-Seq analysis, and multiple cytochrome P450 and carboxylesterase encoding genes were down-regulated in the ME-treated males in both the RNA-Seq and RT-qPCR analyses, with a similar fold change detected. For the other genes tested – *BdorOR43a-1*, *BdorOR43b*, *BdorOR7a-2*, *BdorOR7a-3*, *BdorOR7a-5*, *BdorOR67c*, *BdorOR59a*, and *BdorOR69a* – they expressed no significant differences according to the qRT-PCR test, not unlike the RNA-Seq results. Hence, the qRT-PCR analysis revealed up- or down-regulated gene expression profiles that were consistent with the RNA-Seq data, confirming that our comparative transcriptome analysis was robust and reliable.

### Phylogenetic Analysis of the *BdorORs*

**FIGURE 4:**
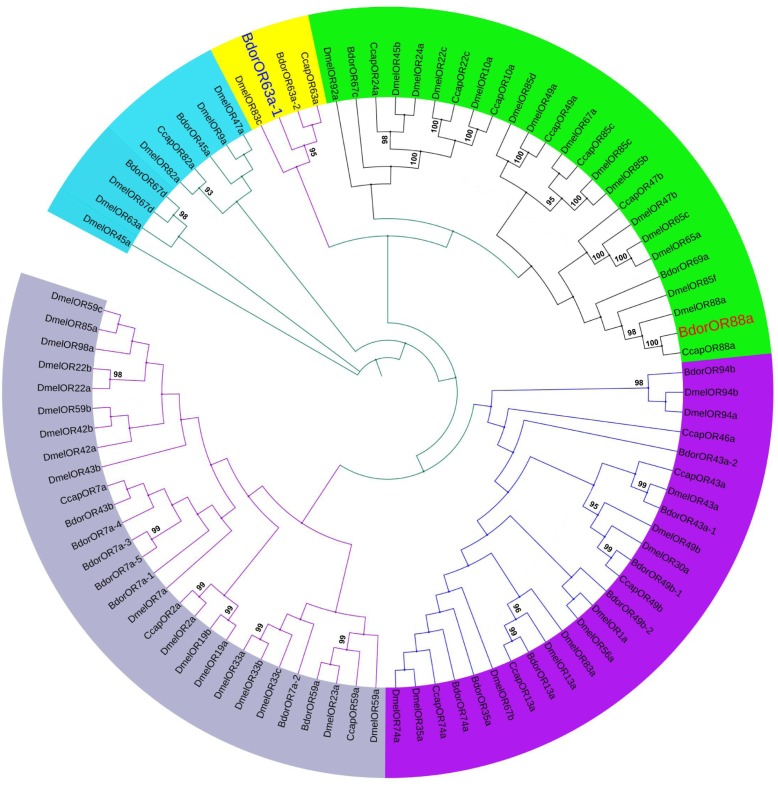
Maximum likelihood dendrogram based on protein sequences of candidate odorant receptors (ORs) in *Bactrocera dorsalis* and other insects. Evolutionary history was inferred using the maximum likelihood method. Bootstrap values greater than 90% (*n* = 1,000 replications) are displayed. ORs from *Bactrocera dorsalis* (Bdor), *Ceratitis capitata* (Ccap), and *Drosophila melanogaster* (Dmel) were included.

The full-length *BdorOR88a* and *BdorOR63a-1* cDNA segments consisted of 1,245 and 1,248 nt, encoding a polypeptide of 414 and 415 amino acids, respectively. To determine the phylogenetic relationship between *BdorORs* and the other ORs reported in *C. capitata* and *D. melanogaster*, a maximum likelihood tree was constructed. The *BdorORs* clustered together with the orthologous ORs from two Dipteran species with the best BLASTP hit. The ligand binding ORs from *B. dorsalis* shared phylogenetic relationships with the OR homologs of both Dipteran species. Notably, *BdorOR88a* clustered in a branch with *DmelOR88a*, but clustered in a different group than *BdorOR63a-1* (**Figure 4**).

### Behavioral Activities of Male Flies in Response to ME and *BdorOR63a-1, BdorOR88a* Expression Level

**FIGURE 5:**
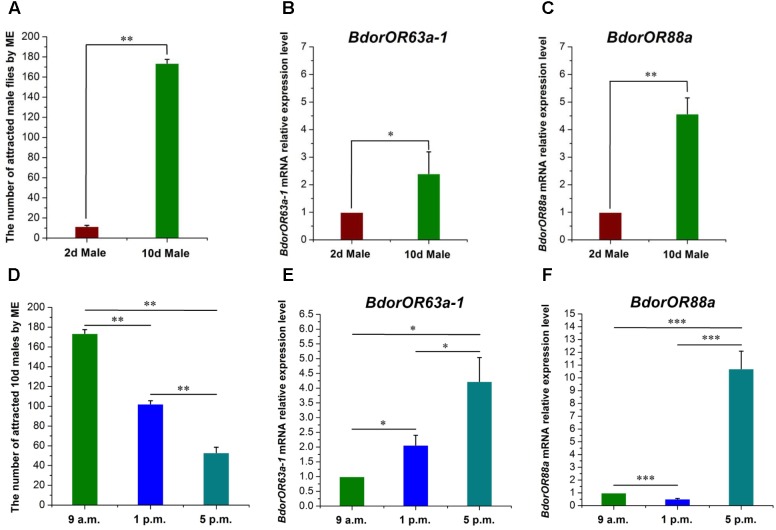
The attractiveness effect of ME (methyl eugenol) to *Bactrocera dorsalis* male flies and the expression pattern of *BdorOR63a-1* and *BdorOR88a* in the males’ antennae. **(A)** Responses of immature and mature male flies to ME. **(B,C)** *BdorOR63a-1* and *BdorOR88a* expressed at higher levels in mature males than immature males antennae. **(D)** Diurnal pattern of mature male responsiveness to ME. **(E,F)** Diurnal pattern of *BdorOR63a-1* and *BdorOR88a* expression levels in the mature males antennae. All the experiments were performed in triplicate. Transcription levels of the *BdorOR63a-1* and *BdorOR88a* gene were normalized by α*-tubulin* gene. The results were evaluated using a 2^-ΔΔC_T_^ method, and the 2^-ΔΔC_T_^ value of calibrant equals to 1.0. Bars represent mean ± SE values. ns, not significant, ^∗^*P* < 0.05, ^∗∗^*P <* 0.01, ^∗∗∗^*P* < 0.001 (*t*-tests).

Under laboratory conditions, the male flies’ taxis to ME had a profile similar to their sexual development. As **Figure 5A** shows, fly responsiveness increased with age, with newly emerged males (2-day-old) presenting the lowest taxis, but by the time they were 10-day-old the males had become highly attracted to ME. The 10-day-old male responders numbered 173.67 ± 3.84, which was about 17 times higher than the abundance of 2-day-old males (11.67 ± 1.00) (*t* = 32.40; *df* = 2; *P* = 0.001). Furthermore, in contrast to the low expression observed in the 2-day-old male flies’ antennae, *BdorOR63a-1* and *BdorOR88a* were highly expressed in 10-day-old males, corresponding to a 2.40-fold (*t* = 5.20; *df* = 2; *P* = 0.035) and 4.57-fold (*t* = 13.01; *df* = 2; *P* = 0.0059) increase, respectively (**Figures 5B,C**).

The diurnal pattern of male responsiveness to ME is illustrated in **Figure 5D**. The male fly response to ME was highest during the morning (173.67 ± 3.84 males), declining to a lower level in the early afternoon (102.33 ± 3.28), but dropped markedly near dusk (53.00 ± 5.51) (*F* = 312.49; *df* = 2; *P* < 0.0001). Furthermore, the expression levels of *BdorOR63a-1* and *BdorOR88a* were not uniform throughout the day. Specifically, the *BdorOR88a* expression level was significantly reduced in the afternoon (p.m.) male adult flies compared with their morning (a.m.) counterparts (*t* = 18.57; *df* = 2; *P* < 0.0001) (**Figure 5F**). Interestingly, the transcript levels of *BdorOR63a-1* and *BdorOR88a* at dusk were dramatically higher than those in the morning and early afternoon males (**Figures 5E,F**).

### Functional Characterization of *BdorOR88a* and *BdorOR63a-1* in the *Xenopus laevis* Oocytes Expression System

**FIGURE 6:**
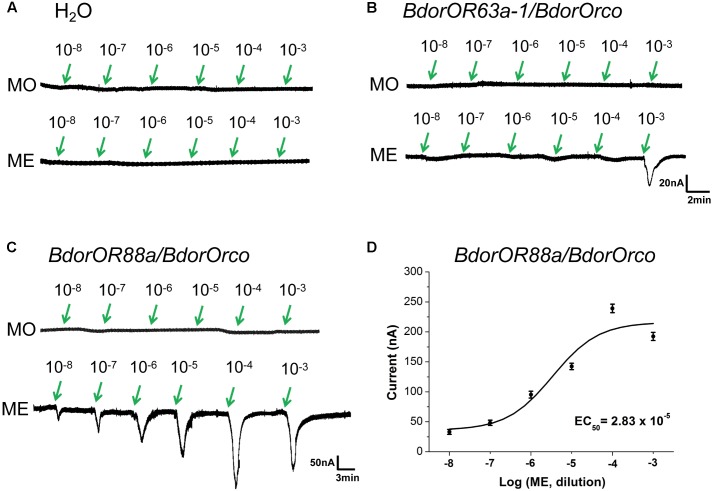
Responses of *Xenopus* oocytes with co-expressed *BdorOR88a*/*BdorOrco* or *BdorOR63a-1/BdorOrco* to stimulation with ME and MO compounds. **(A)** H_2_O-injected *Xenopus* oocytes failed to respond to the ME (methyl eugenol) and MO (mineral oil) odorants. **(B)** *BdorOR63a-1/BdorOrco* only slightly responded to the 10^-3^ M solution of ME. **(C)** *BdorOR88a*/*BdorOrco Xenopus* oocytes were stimulated with a range of ME concentrations. **(D)** Dose-dependent curve of *BdorOR88a* to ME using doses of 1.0 × 10^-8^ to 1.0 × 10^-3^ M. EC_50_ = 2.83 × 10^-5^. Symbols show the electrical current responses from the *BdorOR88a*/*BdorOrco* complex presented as the mean ± SE (*n* = 8). The dose-response curve shown was fitted with a sigmoidal model that had a variable slope (in Origin v9.0 software).

To verify whether ME olfactory detection in the *B. dorsalis* flies is mediated by *BdorOR63a-1* or *BdorOR88a*, we expressed these putative receptors along with co-receptor *BdorOrco*, by using the *Xenopus* oocytes and two-electrode voltage clamping recording system. We found that H_2_O injected oocytes did not generate detectable currents when challenged with either MO or ME (**Figure 6A**). *BdorOR63a-1*/*BdorOrco*-expressed oocytes did not respond to MO and only weakly generated a ∼30 nA current in response to ME, even at concentrations as high as 1 × 10^-3^ M (**Figure 6B**). By contrast, the *BdorOR88a*/*BdorOrco*-expressing oocytes were clearly activated by ME, with no response to MO. Additionally, ME elicited dose-dependent currents from the *BdorOR88a/BdorOrco*-expressing oocytes. This analysis also indicated that the lowest measurable response was observed at a concentration of 1 × 10^-8^ M (**Figure 6C**) and the EC_50_ value – half the maximal effective concentration refers to the concentration of odorant, which induces a response halfway between the baseline and maximum – was 2.83 × 10^-5^ M (**Figure 6D**). Therefore, it is conceivable that *BdorOR88a* is likely to play a role in the reception of ME by *B. dorsalis* males.

### *BdorOR88a* Mediates the Responsiveness of Mature Male Flies to ME

**FIGURE 7:**
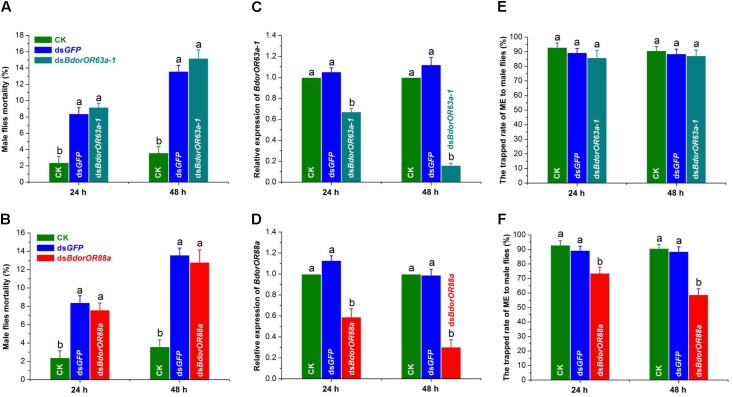
Effects of RNA interference on the mortality of *Bactrocera dorsalis* male flies, *BdorOR63a-1* and *BdorOR88a* expression in their antennae, and fly responsiveness to ME. **(A,B)** Mortality of male flies in the ds*BdorOR63a-1* and ds*BdorOR88a* treatments in the 24 h and 48 h bioassays were calculated. Negative control males were injected with the same amount of ds*GFP*. Blank control groups (CK) were normally reared. **(C,D)** The RNAi efficiency was measured at 24 and 48 h by qRT-PCR after the dsRNA injection. α*-tubulin* gene was used as an internal reference gene. The results were evaluated using a 2^-ΔΔC_T_^ method, and the 2^-ΔΔC_T_^ value of calibrant equals to 1.0. **(E,F)** The attractiveness effect of ME (methyl eugenol) to males after *BdorOR63a-1* and *BdorOR88a* genes were silenced by RNAi. Different letters among the columns within a time point indicate significant differences (ANOVA, *P* < 0.05). Five biological replicates were used. Bars represent mean ± SE values.

Microinjection had a clear and negative influence on *B. dorsalis* mature male flies’ survival. As **Figures 7A,B** show, the average mortalities of flies in the ds*BdorOR63a-1*, ds*BdorOR88a*, and ds*GFP* treatment groups at 24 h were 9.20 ± 0.49, 7.60 ± 0.75, and 8.40 ± 0.75%, respectively, and this mortality increased to 15.20 ± 1.02, 12.80 ± 1.36, and 13.60 ± 0.75% at 48 h (**Figures 7A,B**). However, the mortalities of the blank controls were comparatively much lower: 2.40 ± 0.75% at 24 h (*F* = 16.83; *df* = 3; *P* = 0.0001) and 3.60 ± 0.75% at 48 h (*F* = 24.56; *df* = 3; *P* < 0.0001). Notably, mortality of the ds*BdorOR63a-1*- and ds*BdorOR88a*-treated male flies was not significantly different from that of the ds*GFP*-treated male flies.

Consequently, transcript levels of *BdorOR63a-1* and *BdorOR88a* in the adult males were significantly reduced in the dsRNAs-injected flies compared with the two control groups (**Figures 7C,D**). As **Figure 7C** shows, *BdorOR63a-1* expression was down-regulated in the ds*BdorOR63a-1*-treated males, to a level approximately 1.49-fold lower than that of the blank control and ds*GFP* treatment groups at 24 h (*F* = 55.62; *df* = 2; *P* < 0.0001), a significant difference that reached c. 6.25-fold at 48 h (*F* = 154.08; *df* = 2; *P* < 0.0001). Similarly, after treatment for 24 h and 48 h, there was a pronounced decrease in *BdorOR88a* expression of approximately 1.69-fold (*F* = 31.72; *df* = 2; *P* = 0.0002) and 3.29-fold (*F* = 46.69; *df* = 2; *P* < 0.0001) when compared with that of the ds*GFP* and blank control groups, respectively (**Figure 7D**).

We then analyzed the effects of RNAi of the *BdorOR63a-1* and *BdorOR88a* gene transcript on male responsiveness to ME. Only the *dsBdorOR88a*-treated males were much less trapped by ME than were the ds*GFP*-treated and blank control flies. After 24 h of the treatment, the proportion of males trapped in the ds*GFP*-treated (89.38 ± 2.83%) and blank control (92.97 ± 3.06%) groups were both significantly higher than recorded in the *dsBdorOR88a* treatment (73.60 ± 4.16%; *F* = 53.37; *df* = 2; *P* < 0.0001). Furthermore, this difference was greatest at 48 h, when the proportion of ME-trapped males was 58.80 ± 4.25% in the *dsBdorOR88a* treatment group, significantly less than in the ds*GFP-*treated (88.63 ± 3.13%) and control (90.71 ± 2.82%) groups (*F* = 114.81; *df* = 2; *P* < 0.0001) (**Figure 7F**). By contrast, males were similarly trapped in the *dsBdorOR63a-1* and ds*GFP* treatment groups and the blank control at 24 h (*F* = 2.83; *df* = 2; *P* = 0.1180) and 48 h (*F* = 2.87; *df* = 2; *P* = 0.1151) (**Figure 7E**).

### Hypothesized Modal Analysis of ME Detection and Transportation Process in *Bactrocera dorsalis* Mature Males Antennae

**FIGURE 8:**
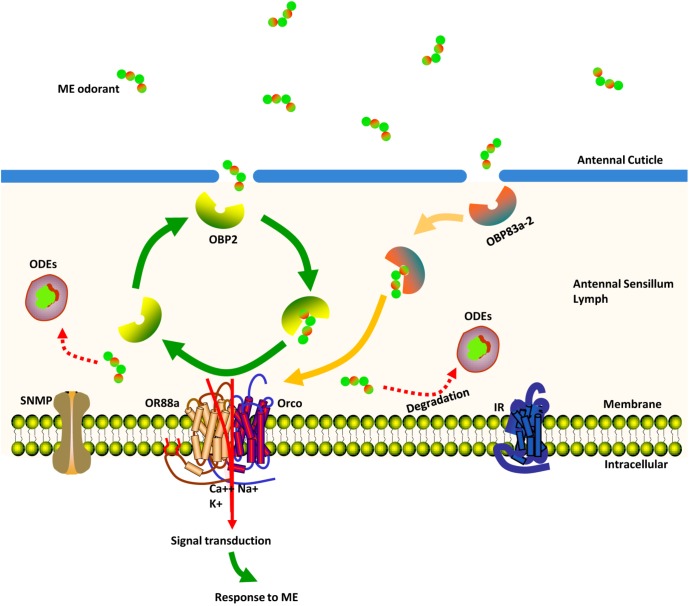
Schematic view of the ME (methyl eugenol) odorant detection process in the antennae of mature *Bactrocera dorsalis* male flies. Once ME odorants penetrate pore tubules of the sensillum, they are bound and solubilized by *BdorOBP83a-2* and *BdorOBP2*, transported through the sensillum lymph and finally reach sensory dendrites, where they activate membrane-bound *BdorOR88a*. Signal transduction evokes a response behavior to the detected ME. ME odorants are rapidly degraded and inactivated by odorant-degrading enzymes.

Here, we propose a model describing how chemosensory proteins might be used by a mature male to distinguish the ME odorant from a noisy environment (**Figure 8**). Once the ME odorant penetrates the pore tubules of the antennae, the protein products of *BdorOBP83a-2* and *BdorOBP2* are posited to assist in transporting the ME odorant across the aqueous sensillum lymph. After its release from these proteins, the ME odorant is then transferred to the protein encoded by *BdorOR88a*. With the ME odorant now bound, the *BdorOR88a*/BdorOrco complexes are activated to trigger the signals that lead to neural spikes generated in the fly’s brain, evoking its characteristic response behavior to ME. After the activation of the odorant receptor, ME odorant is deactivated and degraded by the ODEs.

## Discussion

Because ME is a potent attractant of *B. dorsalis* mature males, this parapheromone has been widely used for decades as a sexual lure in the detection and control of male *B. dorsalis* field populations worldwide (Smith et al., 2002; Shelly et al., 2010; Pagadala et al., 2012). However, until now, we do not know how or why it works. Based on this study’s RNA-Seq and qRT-PCR results, two odorant receptor genes (*BdorOR63a-1* and *BdorOR88a*) were found abundantly expressed in mature males’ antennae after ME stimulation. Next, we functionally characterized these ORs in a heterologous expression system. In contrast to *BdorOR63a-1*, *BdorOR88a* co-expressed with *BdorOrco* in the *Xenopus* oocytes elicited dose-dependent inward currents upon application of ME. Additionally, silencing *BdorOR63a-1* via the injection of dsRNA had no significant effect on the males’ attraction to ME, whereas silencing *BdorOR88a* significantly reduced the number of males attracted to ME. As such, we conclude that *BdorOR88a* is necessary for explaining the observed attraction of mature males toward ME. Hence, our present results further improve the current knowledge of the molecular mechanism underpinning ME detection by *B. dorsalis* mature male flies.

Male fly responsiveness to ME was clearly age-dependent. Males were strongly attracted to ME when 10-day-old, while immature male flies (2-day-old) were not attracted to ME. This result is not unlike that found in prior studies (Karunaratne and Karunaratne, 2012; Liu H. et al., 2017). Accordingly, in contrast to *BdorOR63a-1*, *BdorOR88a* was extreme abundantly expressed in the mature male antennae. ME functions as a precursor for *B. dorsalis* male synthesis of sex pheromone components (Shelly et al., 2008; McInnis et al., 2011; Liu H. et al., 2017). ME-acquired males produced a more attractive sexual pheromonal signal and enjoyed a higher mating success than ME-deprived males (Nishida et al., 1997; Shelly et al., 2000). Decades of publications have suggested that ingestion of ME enhances male mating competitiveness and thus demonstrated that this effect underlies the strong attraction of males to ME (Shelly, 2010). Prior studies had already revealed that male responsiveness to ME was not uniform throughout the day: it peaks in the morning, declines in the afternoon, and drops markedly at dusk (Ibrahim and Hashim, 1980; Tan et al., 1986; Karunaratne and Karunaratne, 2012). Our present study agrees rather well with these observations. The daily fluctuation in male flies responsiveness to ME displays a negative correlation with the daily rhythms of their courting and mating behavior (Karunaratne and Karunaratne, 2012; Liu H. et al., 2017). The attractiveness of a volatile depends on both the chemical properties of volatile and the physiological status of insect (Anton et al., 2007; Gadenne et al., 2016). Generally, responses to sex pheromones are switched on during courting and mating, whereas responses to oviposition-site cues or food odors are switched off. *B. dorsalis* mature males were very active at dusk, but extremely low numbers were attracted to the ME source (Karunaratne and Karunaratne, 2012). During the courting and mating at dusk, *B. dorsalis* males may transiently stop responding to the ME until the next morning and begin responding to sex pheromone in search of a mating partner.

Olfactory plasticity is a powerful evolutionary strategy that optimizes critical resources for insect survival (Gadenne et al., 2016). Recently, study on olfactory plasticity in insects, such as *Agrotis ipsilon*, *Spodoptera littoralis*, *D. melanogaster*, *Anopheles gambiae*, *C. capitata*, and *B. dorsalis*, is becoming one of the hot topics (Jang, 1995; Zhou et al., 2009; Barrozo et al., 2011; Deisig et al., 2012; Rund et al., 2013a,b; Kromann et al., 2015; Jin et al., 2017). Furthermore, olfactory plasticity enables insects to modify their response to semiochemical according to their physiological conditions, such as, feeding state, circadian rhythm, age, and mating status (Jin et al., 2017). Interestingly, olfactory-guided behaviors vary according to the time of day, which help insects respond to environmental chemical stimulus at the right moment (Gadenne et al., 2016). Considerable literature indicated that antennal and olfactory sensory neurons (OSNs) sensitivity of many insects tends to be regulated by the body endogenous rhythm or biological clock (Krishnan et al., 1999; Page and Koelling, 2003; Tanoue et al., 2004; Jin et al., 2017). Clock genes in the *Drosophila* antennae allow autonomous rhythmicity of environmental cues detection (Krishnan et al., 1999; Tanoue et al., 2004). In *Anopheles gambiae* mosquitoes, OBPs gene expression rhythms are driven in part by the endogenous circadian clock (Rund et al., 2013a,b). The chemosensory receptors gene expression levels of *B. dorsalis* gravid female flies fluctuate rhythmically at different times of the day (Jin et al., 2017). In our study, the expression level of *BdorOR63a-1* increased gradually from morning to dusk in the mature male antennae. Nonetheless, *BdorOR88a* was abundantly expressed during the morning, but much less so in the afternoon. Yet more remarkably, *BdorOR88a* expression level increased considerably at the dusk period. *B. dorsalis* adults mating activity is restricted to dusk. The accurate detection and recognition of a potential mating partner is the key step in insect courtship and subsequently mating (Sayin et al., 2018). The peak in expression of *BdorOR63a-1* and *BdorOR88a* at dusk may correspond to the time of increasing male chemosensory activity to female-emitted sex pheromone blend (containing several components).

Comparative phylogenetic analyses of the OR repertoire of insects can provide useful information on the evolutionary origin of OR families and their expansion in insect lineages (Missbach et al., 2014; Koenig et al., 2015). More strikingly, in our phylogenetic analysis, *BdorOR88a* was distributed within a distinct cluster of ORs with *DmelOR88*a, suggesting that they probably perform the same molecular function in the neuronal circuitry. Intriguingly, in *Drosophila*, the olfactory receptor OR88a attuned to semiochemicals has been identified. OSNs expressing the olfactory receptor OR88a housed in trichoid sensilla of *Drosophila* antennae can mediate responses to unidentified odors in the fly’s male and female body wash extracts (van Naters and Carlson, 2007). More recently, *D. melanogaster* OR88a has been characterized as a receptor of the fly-produced odorants methyl myristate, methyl palmitate, and methyl laurate that mediated copulation and attraction (Dweck et al., 2015). Analogous to other fly OR types, the role of *DmelOR88a* has been considered that of a pheromone receptor (PR) (Dweck et al., 2015; Fleischer et al., 2017). Therefore, we strongly suspect that *B. dorsalis* mature males’ possible use *BdorOR88a* to detect female sex-pheromones and locate their mates at dusk, although further functional experiments are needed to confirm this hypothesis.

From chemical stimulus to behavioral response, the olfactory process involves the capture, binding, transport, and inactivation of odors. The initial steps in odor detection involve the binding of an odor to the ORs positioned on the dendritic membrane of the OSNs within antennae. OBPs are able to bind various hydrophobic odorant molecules in the environment (Sato et al., 2008; Taylor et al., 2008; Silbering et al., 2011; Siciliano et al., 2014). Once semiochemicals are bound, the OR/Orco heteromeric complexes are activated to trigger signals leading to characteristic spikes generated in the brain, thereby producing a behavioral response in the insect (Neuhaus et al., 2005; Benton et al., 2006; Hallem and Carlson, 2006; Sato et al., 2008; Croset et al., 2010; Silbering et al., 2011; Di et al., 2017; Fleischer et al., 2017). Gene silencing, via the ingestion or microinjection of dsRNA, recently confirmed that *BdorOrco*, *BdorOBP83a-2*, *and BdorOBP2* actively had critical roles in mediating the taxis of *B. dorsalis* males to ME (Zheng et al., 2012; Wu et al., 2016; Liu H. et al., 2017). Considering our results alongside those of prior research to date, we preliminarily posit that the ME odor molecules bind to either *BdorOBP2* or *BdorOBP83a-2*, after which they are transferred to OSNs, where *BdorOR88a* coordinates with *BdorOrco* to bind to the ME odor molecules – only then is the olfactory signal transduction pathway finally activated. Our findings from this study thus represent an important breakthrough in our mechanistic understanding of how *B. dorsalis* mature male flies are attracted to ME. Hopefully, this will advance how attractants are designed to target this specific olfactory pathway, which should promote the development of better and more affordable attractants for *B. dorsalis* management in the field.

Combinatorial coding, that is individual OR can be activated by multiple odorants and a specific odor ligand can be detected by multiple ORs, is the primary coding mode of the insect olfaction system (Malnic et al., 1999; Hallem et al., 2004; Suh et al., 2014; Andersson et al., 2015; Fleischer et al., 2017). In *D. melanogaster*, olfactory system sensitivity seems to be enhanced by a combinatorial coding, with specific groups of ORs detecting the same chemical cues (Leal, 2013). Yet more remarkably, knock-down *BdorOR88a* gene did not lead to the complete disappearance of the mature male flies’ responsiveness to ME. Therefore, presumably, in addition to *BdorOR88a*, there are further ORs and other receptors, which may also contribute to detect ME. However, due to the lack of *B. dorsalis* genomic information, many genes cannot yet be annotated and their functions remain unknown, especially for those olfactory-related genes. In their detection of odorant signals, insects use several families of chemosensory receptors, including the ORs, ionotropic receptors (IRs), gustatory receptors (GRs), and sensory neuron membrane proteins (SNMPs) (Benton et al., 2007, 2009; Jin et al., 2008; Suh et al., 2014). The identification of a new family of IRs, complementary to the ORs family yet expressed in different olfactory neurons, has provided new insight into the molecular mechanisms of odor detection in insect (Benton et al., 2009; Silbering et al., 2011). IR92a mediates attraction behavior to ammonia and volatile amines in *D. melanogaster* (Min et al., 2013). SNMPs are highly conserved in multiple insect species and involved in pheromone-based chemical communication (Nichols and Vogt, 2008). One subfamily in particular, SNMP1, when co-expressed with PRs, is believed to contribute to the sensitivity of pheromone detection in insects (Rogers et al., 1997; Benton et al., 2007; Vogt et al., 2009; Liu et al., 2014; Pregitzer et al., 2014). Study has demonstrated that SNMP1 played a vital role in detecting the sex pheromone Z11-18OAc in *D. melanogaster* (Benton et al., 2007; Jin et al., 2008). Accordingly, in moths, SNMP1 is considered indicative of sex pheromone-responsive neurons in antennae (Forstner et al., 2008; Thode et al., 2008). In our present study, the consistency between the RNA-Seq results and the mRNA expression from the qRT-PCR analysis implies that *BdorIR92a* and *BdorSNMP1-1* possibly participate in the processing of ME detection by mature *B. dorsalis* male flies. Nonetheless, knowledge of the precise functional relevance of *BdorIR92a* and *BdorSNMP1-1* for ME signaling remains elusive. To conclude, we deduce that these genes are involved in how *B. dorsalis* males detect ME, but this awaits further investigation and testing.

## Author Contributions

HL and Y-YL conceived and designed the experiments and wrote the manuscript. HL, Z-SC, and D-JZ performed the experiments. HL analyzed the data. Z-SC and D-JZ provided material support. All authors discussed the results and reviewed the final manuscript.

## Conflict of Interest Statement

The authors declare that the research was conducted in the absence of any commercial or financial relationships that could be construed as a potential conflict of interest.

## Footnotes

1 http://www.geneontology.org

2 http://www.genome.jp/kegg/

3 http://www.ncbi.nlm.nih.gov/COG/

4 http://www.ebi.ac.uk/uniprot/

5 http://www.blast2go.com/

6 http://itol.embl.de
