# Supplementary material for: BdorOR88a Modulates the Responsiveness to Methyl Eugenol in Mature Males of Bactrocera dorsalis (Hendel)
Source: Front Physiol. 2018 Jul 26;9:987. doi: 10.3389/fphys.2018.00987 (PMC6094957; doi:10.3389/fphys.2018.00987)
Supplement: Supplementary file 1 [file Presentation_1.ZIP › Supplementary materials--revision/Supplementary Figure S1-S2.docx]

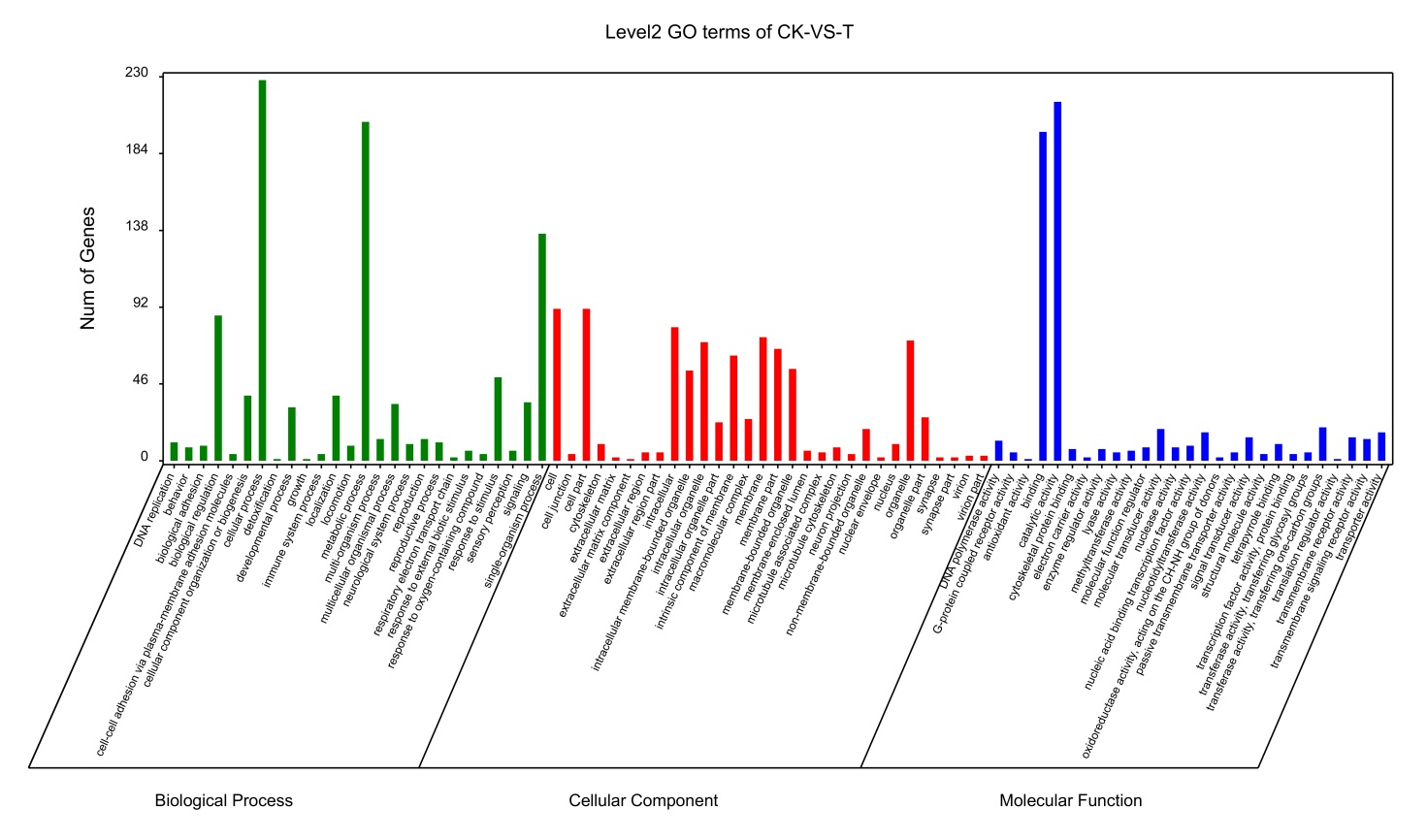


**Figure S1. Gene Ontology classifications of antennal differentially expressed genes as identified by RNA-seq in ME and MO treatment *Bactrocera dorsalis* male files.**


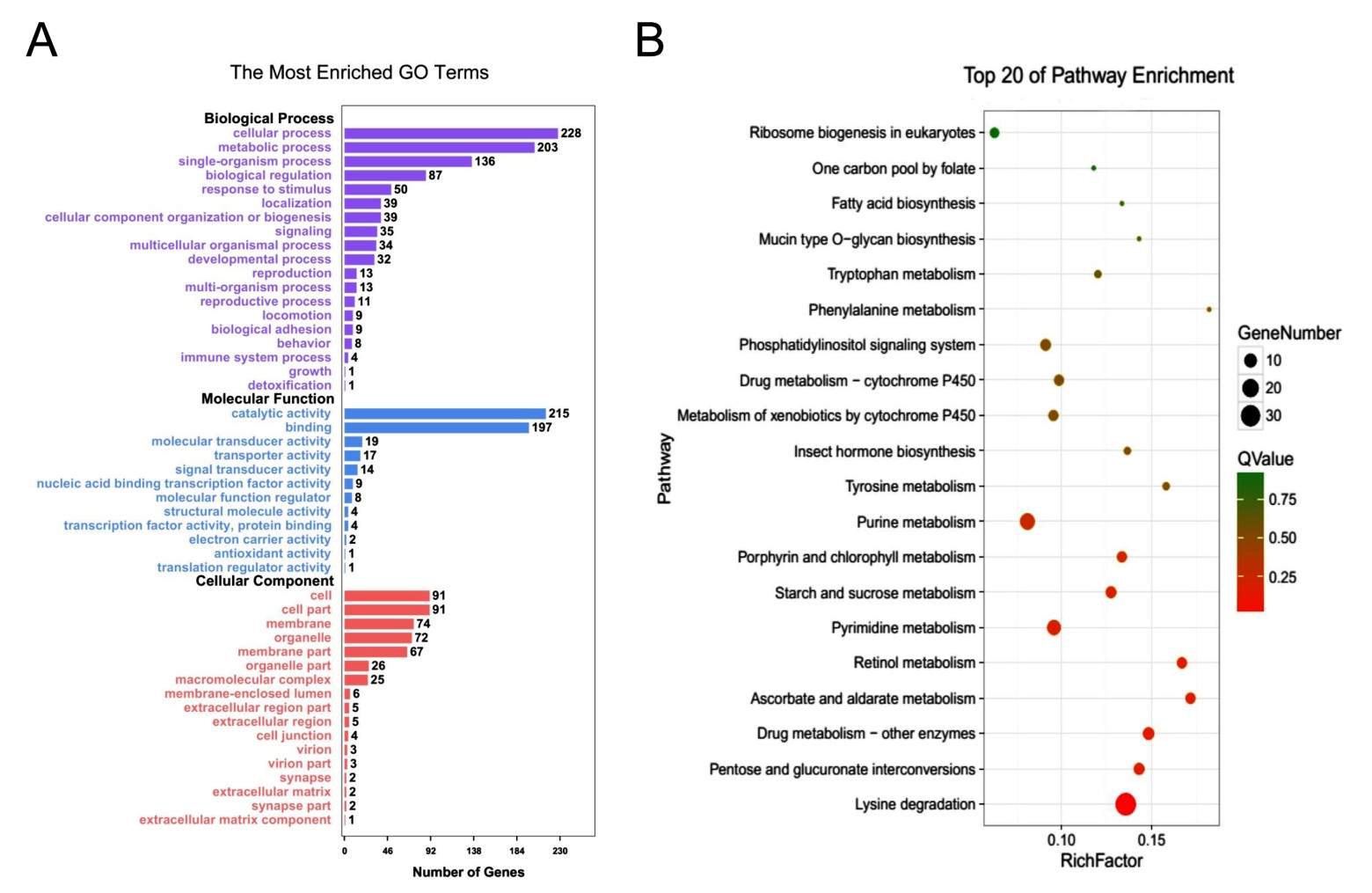


**Figure S2. Significantly enriched GO categories and KEGG pathways in the DEGs of *Bactrocera dorsalis* male antennae from the ME (methyl eugenol) and MO (mineral oil) treatment groups.** (A) Results of the GO enrichment analysis of differentially expressed genes (DEGs). The significantly enriched GO terms (corrected *P*-value < 0.05) were divided into three main categories: molecular function, cellular component, and biological process. The *x*-axis represents the number of DEGs and *y*-axis represents the enriched GO terms. (B) KEGG significant enrichment analysis for those genes differentially expressed between the ME and MO males. The color and size of the dots represent the range of Q values (corrected *P* value) and gene number, respectively.
