# Supplementary material for: BdorOR88a Modulates the Responsiveness to Methyl Eugenol in Mature Males of Bactrocera dorsalis (Hendel)
Source: Front Physiol. 2018 Jul 26;9:987. doi: 10.3389/fphys.2018.00987 (PMC6094957; doi:10.3389/fphys.2018.00987)
Supplement: Supplementary file 1 [file Presentation_1.ZIP › Supplementary materials--revision/Supplementary Table S1-S3.docx]

**Table S1.** Primers used for gene expression detection by qPCR analysis.

| **Primer name** | **Nucleotide sequences (Forward)** | **Nucleotide sequences (Reverse)** | **Primer efficiency (%)** |
| --- | --- | --- | --- |
| *Carboxylesterase* | CACTAAAGAAGCCAGCGATG | TTATAAGTGGTAAGGAGAAG | 91.88 |
| *Cytochrome P450* | CCACTAATGACTGAGATCGG | AGACTGTTGGCTTTCACACC | 92.08 |
| *BdorOR43a-1* | GCTCTTCACCTATTACTGGC | GCATCTGACCAACGCGGATC | 96.73 |
| *BdorOR43b* | AGCAGGTGGTGACGGTAAC | TGTCCTCCTGTGCACGATG | 97.24 |
| *BdorOR7a-2* | CCGCACGGAGTTTGTAATTG | TCTACCTGCGTTAGTGTTGG | 92.83 |
| *BdorOR7a-3* | CAATTCACTGTCTATGCGGC | GAAGCTTGATAACAGGTCGG | 93.11 |
| *BdorOR7a-5* | GACAATGGACTCGTTACCTG | GTGAGAGAAAGGATCTTGCC | 92.53 |
| *BdorOR67c* | TCAACCGTTAACTTATGCCG | CCAGCGTCCTCATATCAGC | 92.45 |
| *BdorOR59a* | TTAACGCGTCCACCTCCAG | CTGCAGACCACACAGATAAC | 95.24 |
| *BdorOR69a* | CCTACTTCACCTTGGATCTG | GGTTACCTGCACGATCGTAG | 93.43 |
| *BdorOBP57c* | GTATTTGCGTTGCCACCTGG | GCCATTTAGCAGACAATCGG | 92.35 |
| *BdorOBP5* | CAAGGAGCACAATGTATCGC | CATTCATCCACAGCAGCAAC | 97.84 |
| *BdorOR63a-1* | CTGCTACAACAGGTGATTGC | GTAGCCAAGGACATTACTGTG | 95.61 |
| *BdorOR88a* | TGTATGCTTCGTGGTTACCG | CATCCGGCACATTCATTTCC | 96.87 |
| *BdorIR92a* | GTCAGTCAACTGGATGTCGG | GAAACGTACTGTGTCCGAAAG | 93.42 |
| *BdorSNMP1-1* | CAGATCCGAGCTTGCATTGC | AACACCGCATGATCCTTCTC | 94.12 |
| *α-tubulin* | CGCATTCATGGTTGATAACG | GGGCACCAAGTTAGTCTGGA | 94.73 |

**Table S2.** Primers used for two-electrode voltage-clamp electrophysiological recordings.

| **Primer name** | **Nucleotide sequences (Forward)** | **Nucleotide sequences (Reverse)** |
| --- | --- | --- |
| *BdorOR63a-1* | CGGAATTCGCCACCATGTACAGCATAAGTGAAATA | CGGCTCGAGTTAAGTTTCATCAATATCTCGAAGT |
| *BdorOR88a* | CGGAATTCGCCACCATGGCGCCGCAACAGGAAGTG | CGGCTCGAGTCATTTTGTGTAACCCTTTGCCTTC |
| *BdorOrco* | CGGAATTCGCCACCATGCAGCCCAGCAAATATGTG | CGGCTCGAGCTACTTCAATTGCACCAGCACCATG |

**Table S3.** Primers used for RNA interference analysis.

| **Primer name** | **Nucleotide sequences (Forward)** | **Nucleotide sequences (Reverse)** |
| --- | --- | --- |
| *dsOBP63a-1* | TAATACGACTCACTATAGGGAGACCACTCCAGGTGTTATGTCTACTG | TAATACGACTCACTATAGGGAGACCACGTAGCCAAGGACATTACTGT |
| *dsOR88a* | TAATACGACTCACTATAGGGAGACCACTGTATGCTTCGTGGTTACCG | TAATACGACTCACTATAGGGAGACCACCATACCACTGCTGTTCGTAG |
| *dsGFP* | TAATACGACTCACTATAGGGGAGACCACACGGCCACAAGTTCAGCGT | TAATACGACTCACTATAGGGGAGACCACGACCACTACCAGCAGAACA |

Note: The underlined nucleotides sequence is T7 promoter.
